# Efficacy and safety of intermittent theta burst stimulation versus high-frequency repetitive transcranial magnetic stimulation for patients with treatment-resistant depression: a systematic review

**DOI:** 10.3389/fpsyt.2023.1244289

**Published:** 2023-07-31

**Authors:** Xian-Jun Lan, Xin-Hu Yang, Zhen-Juan Qin, Dong-Bin Cai, Qi-Man Liu, Jian-Xin Mai, Can-jin Deng, Xing-Bing Huang, Wei Zheng

**Affiliations:** ^1^The Brain Hospital of Guangxi Zhuang Autonomous Region, Liuzhou, China; ^2^The Affiliated Brain Hospital of Guangzhou Medical University, Guangzhou, China; ^3^Shenzhen Traditional Chinese Medicine Hospital, Shenzhen, China

**Keywords:** intermittent theta burst stimulation, high-frequency rTMS, treatment-resistant depression, systematic review, response

## Abstract

**Objective:**

Intermittent theta-burst stimulation (iTBS), which is a form of repetitive transcranial magnetic stimulation (rTMS), can produce 600 pulses to the left dorsolateral prefrontal cortex (DLPFC) in a stimulation time of just over 3 min. The objective of this systematic review was to compare the safety and efficacy of iTBS and high-frequency (≥ 5 Hz) rTMS (HF-rTMS) for patients with treatment-resistant depression (TRD).

**Methods:**

Randomized controlled trials (RCTs) comparing the efficacy and safety of iTBS and HF-rTMS were identified by searching English and Chinese databases. The primary outcomes were study-defined response and remission.

**Results:**

Two RCTs (*n* = 474) investigating the efficacy and safety of adjunctive iTBS (*n* = 239) versus HF-rTMS (*n* = 235) for adult patients with TRD met the inclusion criteria. Among the two included studies (Jadad score = 5), all were classified as high quality. No group differences were found regarding the overall rates of response (iTBS group: 48.0% versus HF-rTMS group: 45.5%) and remission (iTBS group: 30.0% versus HF-rTMS group: 25.2%; all *Ps* > 0.05). The rates of discontinuation and adverse events such as headache were similar between the two groups (all *Ps* > 0.05).

**Conclusion:**

The antidepressant effects and safety of iTBS and HF-rTMS appeared to be similar for patients with TRD, although additional RCTs with rigorous methodology are needed.

## Introduction

Depression is a leading cause of disability worldwide and a major contributor to the global burden of disease; it is estimated to be the strongest contributor among developed countries by the end of 2030 (1). Major depressive disorder (MDD) has an estimated lifetime prevalence of 3.4% and a 12-month prevalence of 2.1% according to the latest national epidemiological survey from China (2). Over 700,000 people die by suicide every year, and more than half of these deaths are caused by depression (3). Currently, traditional treatments for MDD include antidepressant medication and psychotherapy, but more than one-third of patients fail to respond to either pharmacotherapy or psychotherapy (4–6). Similarly, up to 30% of patients do not achieve clinical remission (7, 8). In addition, multiple side effects of medication could lead to a poor quality of life and reduced treatment adherence (9). There is still a lack of effective strategies for addressing treatment-resistant depression (TRD). Therefore, new treatment modalities for patients with TRD are urgently needed.

Noninvasive brain stimulation techniques, such as transcranial magnetic stimulation (TMS) (10), transcranial direct current stimulation (tDCS) (11), and transcranial alternating current stimulation (tACS) (12), provide a nonpharmacological alternative for MDD. High-frequency (≥ 5 Hz) repetitive transcranial magnetic stimulation (HF-rTMS) was approved by the Food and Drug Administration (FDA) as a noninvasive brain stimulation technique for TRD in 2008 (13). Evidence for the supremacy of active rTMS over sham stimulation has been accumulating for nearly 20 years (10, 14). A recent study analyzing 81 randomized clinical trials (RCTs) found that active rTMS targeting the left dorsolateral prefrontal cortex (DLPFC) led to a higher rate of clinical remission and response compared to sham stimulation (15). However, a retrospective study found that only 214/730 depressed patients (29.3%) obtained antidepressant response to HF-rTMS, showing that not all patients with MDD could benefit from HF-rTMS (16). In particular, the antidepressant effects of rTMS were not evident in patients with high resistance to prior antidepressant treatments (17). Given that the standard FDA-approved HF-rTMS protocol requires 37.5 min per session and a long treatment course (5 times per week and lasting 4–6 weeks) (18), this approach may increase the daily transport burden and inconvenience for full-time patients, thereby reducing the clinical feasibility of conventional rTMS (19).

New efficient strategies for enhancing the therapeutic efficiency of rTMS are a hot topic in current research and have shown significant clinical value. As a novel and potentially beneficial form of TMS, theta-burst stimulation (TBS) including continuous TBS (cTBS), intermittent TBS (iTBS), bilateral TBS (bTBS), and intermediate TBS (imTBS) have been popularly used in clinical practice (20). Notably, iTBS can produce 600 pulses in a total stimulation time of 3 min 9 s (20), which was also approved by the FDA in 2018 for the treatment of TRD (21). Previous pilot studies have shown that active iTBS is superior to sham stimulation for TRD (22–24). A retrospective study initially investigating the antidepressant outcomes of iTBS versus HF-rTMS over the left DLPFC found that 3-min iTBS protocols may be as effective as HF-rTMS protocols (25). Two randomized controlled studies (RCTs) consistently reported similar antidepressant effects and safety with iTBS and HF-rTMS as an adjunctive treatment for patients with TRD (26, 27). For example, Blumberger et al. carried out a large multicentre RCT that confirmed that iTBS over the left DLPFC as an add-on therapy was noninferior to HF-rTMS as measured by the Hamilton Rating Scale for Depression (HRSD) for the treatment of patients with TRD (26). Similarly, a recently published study showed similar response rates (36.7% versus 33.3%) and remission rates (18.5% versus 14.8%) as evaluated by the Montgomery-Åsberg Depression Rating Scale (MADRS) in patients suffering from TRD treated with iTBS and HF-rTMS (27). The 3-min iTBS protocol seems to be an optimized solution for reducing depressive symptoms, as it saves time and improves acceptability in the treatment of TRD when compared to traditional HF-rTMS.

To date, no systematic review investigating the safety and antidepressant effects of iTBS versus HF-rTMS were published. To fill this gap, we performed this systematic review to evaluate the efficacy and safety of iTBS versus HF-rTMS in the treatment of patients with TRD. Based on the findings of Mutz et al.’s study (28), we hypothesized that iTBS has a similar antidepressant effect as HF-rTMS in adult patients with TRD.

## Methods

### Search strategy and screening criteria

Two researchers (X-JL and Z-JQ) systematically searched the Cochrane Library, PubMed, EMBASE, PsycINFO, Chinese Journal Net, and WanFang databases from inception to 19 November 2022 to identify relevant studies using the following search terms: “(“intermittent theta-burst stimulation” OR (intermittent* AND “theta-burst stimulation”) OR iTBS)” AND (trans-cranial magnetic stimulation OR transcranial magnetic stimulation OR rTMS OR TMS) AND (depress* OR dysphor* OR melanchol* OR antidepress*). Additionally, the references of identified RCTs (26, 27) and relevant articles (29, 30) were manually searched to identify missing studies on the safety and efficacy of iTBS versus HF-rTMS for TRD.

As recommended by the Preferred Reporting Items for Systematic Reviews and Meta-Analyzes (PRISMA) guidelines (31), any published RCTs comparing iTBS and HF-rTMS for TRD were included when they met the following inclusion criteria, which were developed based on the *PICOS* principles: *P*articipants: adult patients (more than 18 years) with a primary diagnosis of TRD defined by the respective studies; *I*ntervention: treatments as usual (TAU) plus active iTBS; *C*omparison: TAU plus HF-rTMS (≥ 5 Hz); *O*utcomes: the primary outcomes of interest were the study-defined response and study-defined remission as measured by HRSD or MADRS; secondary results were the rates of discontinuation and adverse events; *S*tudy: only published RCTs comparing the safety and efficacy of iTBS and HF-rTMS for patients with TRD were eligible for inclusion in this systematic review. Numerous studies have found that a standard run of iTBS (600 pulses/session) presents similar or more potent excitatory effects in brain regions than conventional rTMS (32–34). As recommended previously (20, 26), the 3-min protocol of iTBS has a unique advantage in reducing treatment time. Thus, only studies examining daily treatment using a standard dose of 600 pulses of iTBS were included. Studies focusing on other modalities of iTBS, such as accelerated iTBS (≥2 sessions/day) (35) and prolonged iTBS (1800 pulses per session) (36), were excluded. Review articles, meta-analyzes, and case reports or case series were also excluded.

### Data extraction

Data extraction for each included RCT was conducted by two independent researchers (X-JL and Z-JQ) using a standardized Microsoft Excel sheet, focusing on the following subjects: study design, participant characteristics, parameters of iTBS and HF-rTMS, and treatment outcomes from the original research. Any differences in data entry between the two researchers (X-JL and Z-JQ) were discussed with a senior author (D-BC), if necessary. For the missing information or clarification, we would contact the author(s) by email or telephone.

### Study quality assessment

Two researchers (X-JL and Z-JQ) independently assessed the quality of the included RCTs using the Jadad scale (37) and Cochrane risk of bias tool (38). RCTs with a Jadad score ≥ 3 were considered to be of high quality (39). In addition, the overall evidence level and strength for all primary and secondary outcomes were rated by using the Grading of Recommendations Assessment, Development, and Evaluation (GRADE) system (40).

## Results

### Literature search

**Figure 1:**
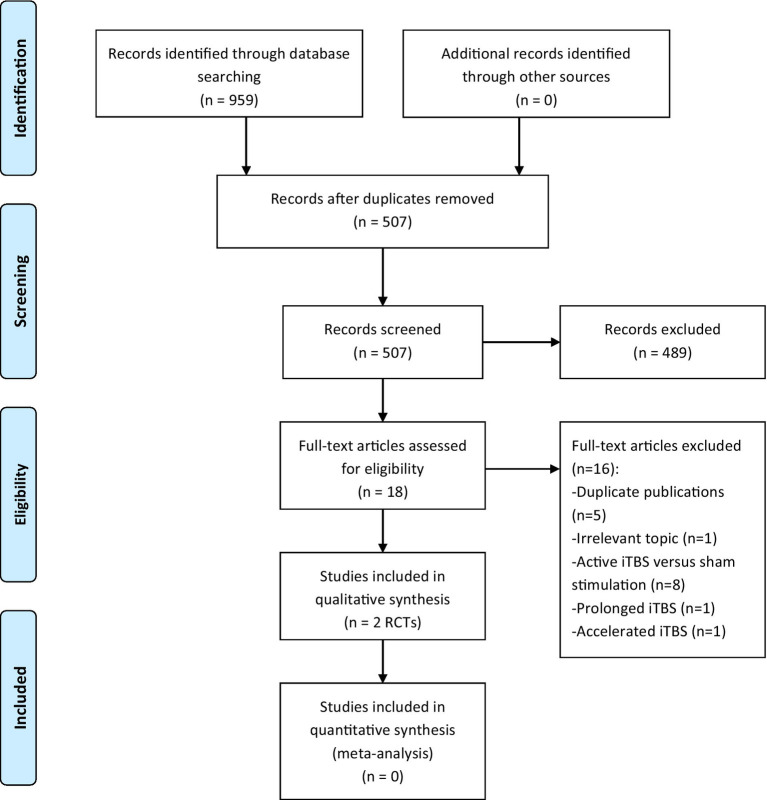
PRISMA flow diagram. iTBS, intermittent theta burst stimulation; PRISMA, Preferred Reporting Items for Systematic Reviews and Meta-analyzes; RCTs, randomized controlled trials.

We initially retrieved 959 articles by searching the above databases. Ultimately, 2 RCTs (26, 27) met the inclusion criteria of the present systematic review. The study selection process is presented in Figure 1.

### The characteristics of the included studies

Table 1 provides a summary of clinical characteristics and the detailed treatment protocols for each included RCT (26, 27). Two RCTs (*n* = 474) compared the efficacy and safety of iTBS (*n* = 239) and HF-rTMS (*n* = 235) for adult patients with TRD. In the two RCTs, the dose of iTBS (50 Hz) was 600 pulses per session, and the doses of HF-rTMS ranged from 1,600 to 3,000 pulses per session. Participants in iTBS groups experienced a total dose of 12,000 pulses in both RCTs, and the total dose of HF-rTMS varied from 32,000 to 60,000 pulses. Their mean duration of illness ranged from 19.5 to 23.3 months, and the proportion of male patients with TRD was between 31.7% and 40.6%. The treatment duration in both studies was 20 days.

**Table 1:** Participant characteristics and HF-rTMS/iTBS parameters of each included study in this systematic review.

Study (country)	Sample size (n)^a^	-Diagnostic criteria-setting (%)-diagnosis (%)	-Illness duration (months)-male^a^ (%)	Mean age^a^ (range)	Medication status	-Treatment duration (days)-site (rTMS/iTBS)	-Intensity (%RMT)-frequency (Hz)		-Stimulus time/per session-train duration-intertrain duration		-Pulses per session-number of sessions-total pulses		Jadad score
							iTBS	HF-rTMS	iTBS	HF-rTMS	iTBS	HF-rTMS	
Blumberger et al. (26) (Canada)	Total: 414 iTBS: 209 HF-rTMS: 205	-DSM-IV-TR or ICD-10^b^ -NR -TRD (100)	−23.3 −40.6	42.4 (18–65) years	Psychotropic allowed	−20 -L-DLPFC	−120 −50	−120 −10	−3 min 9 s −2 s −8 s	−37.5 min −4 s −26 s	−600 −20 −12,000	−3,000 −20 −60,000	5
Bulteau et al. (27) (France)	Total: 60 iTBS: 30 HF-rTMS: 30	-DSM-5 -In (5) and outpatients (95) -TRD (100)	−19.5 −31.7	52.3 (18–75) years	Psychotropic allowed	−20 -L-DLPFC	−80 −50	−110 −10	-NR -NR -NR	−20 min −4 s −28 s	−600 −20 −12,000	−1,600 −20 −32,000	5

a Overall number of participants.

b Diagnosis verified through the Mini International Neuropsychiatric Interview (MINI).

DSM-5, Diagnostic and Statistical Manual of Mental Disorders 5th edition; DSM-IV-TR, Diagnostic and Statistical Manual of Mental Disorders 4th edition, text revision; HF-rTMS, high-frequency repetitive transcranial magnetic stimulation; ICD-10, International Classification of Diseases, 10th edition; iTBS, intermittent theta burst stimulation; L-DLPFC, left dorsolateral prefrontal cortex; min, minutes; NR, not reported; rTMS, repetitive transcranial magnetic stimulation; RMT, resting motor threshold; s, seconds; TRD, treatment-resistant depression.

### Study quality assessment

**Figure 2:**
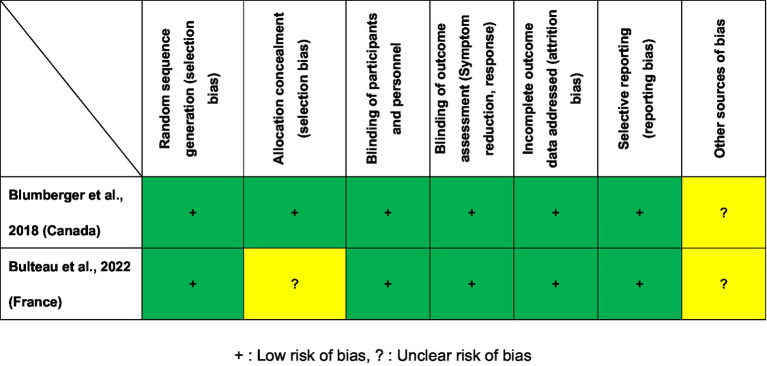
Cochrane risk of bias. +, Low risk of bias; ?, Unclear risk of bias.

Figure 2 presents the Cochrane risk of bias for the two included RCTs. Two RCTs (26, 27) were judged to be low risk regarding selection bias, blinding, attrition and reporting bias. As shown in Table 1, the Jadad scores of the two studies (26, 27) were 5 points (high quality). On the basis of the GRADE guidelines, the overall evidence level for the 17 primary and secondary outcomes of the two included RCTs (26, 27) ranged from “moderate” (5.9%, 1/17) to “high” (94.1%, 16/17; Supplementary Table S2).

### Primary outcomes

As shown in Table 2, two RCTs (26, 27) reported the rates of study-defined remission and response at the intervention endpoint. Among the two RCTs, no group differences were found regarding the overall rates of response (iTBS group: 48.0% versus HF-rTMS group: 45.5%) and remission (iTBS group: 30.0% versus HF-rTMS group: 25.2%; all *Ps* > 0.05).

**Table 2:** iTBS versus HF-rTMS for patients with TRD: study-defined response and remission.

Study	Treatment outcomes	iTBS group	HF-rTMS group	Findings^e^
Blumberger et al. (26) (Canada)	Study-defined response^a^	49.2% (95/193)	47.4% (91/192)	*p* > 0.05
Bulteau et al. (27) (France)	Study-defined response^b^	36.7% (12/30)	33.3% (10/30)	*P* > 0.05
	Total	48.0% (107/223)	45.5% (101/222)	*P* > 0.05
Blumberger et al. (26) (Canada)	Study-defined remission^c^	31.6% (61/193)	26.6% (51/192)	*P* > 0.05
Bulteau et al. (27) (France)	Study-defined remission^d^	18.5% (6/30)	14.8% (5/30)	*P* > 0.05
	Total	30.0% (67/223)	25.2% (56/222)	*P* > 0.05

a Defined as ≥ 50% reduction from the HRSD total score at baseline.

b Defined as ≥ 50% reduction from the MADRS total score at baseline.

c Defined as HRSD scores < 8.

d Defined as MADRS scores < 8.

e Reflect the differences between iTBS groups and HF-rTMS groups at the treatment endpoints.

HF-rTMS, high-frequency repetitive transcranial magnetic stimulation; HRSD, Hamilton Rating Scale for Depression; iTBS, intermittent theta burst stimulation; MADRS, Montgomery-Åsberg Depression Rating Scale; TRD, treatment-resistant depression.

### Secondary outcomes

No group differences were found in terms of discontinuation rates (iTBS group: 7.9% versus HF-rTMS group: 6.8%) or adverse events (e.g., headache, dizziness, nausea, and fatigue) in the two included RCTs (26, 27) (all *Ps* > 0.05). Details are presented in Supplementary Table S1.

## Discussion

To the best of our knowledge, this study is the first systematic review of RCTs to investigate the efficacy and safety of iTBS versus HF-rTMS for patients suffering from TRD. As a result, only two RCTs (26, 27) involving 474 subjects were included. The two included RCTs were published within the last 5 years, suggesting that iTBS and HF-rTMS for subjects suffering from TRD is a new and clinically important topic. The following two major findings of this systematic review included: (1) the antidepressant effects of iTBS and HF-rTMS for patients with TRD were equivalent, and (2) iTBS using 600 pulses per session for patients with TRD among adults was relatively safe and well tolerated.

As reported in this systematic review, the two included RCTs (26, 27) used a standard operation of 600 pulses of unilateral iTBS over the left DLPFC for adult patients with TRD and achieved a similar rate of antidepressant response and remission when compared to HF-rTMS. One RCT (27) examining the long-term effectiveness of iTBS versus HF-rTMS in patients with TRD found that both groups had a similar significant improvement of depressive symptoms at 6 months. Similarly, a large network meta-analysis (113 trials, 6,750 participants) found that iTBS was superior to sham stimulation and had similar antidepressant effects as conventional rTMS (including HF-rTMS, low-frequency rTMS, and bilateral rTMS) (28). Interestingly, a similar antidepressant efficacy between intensive/accelerated iTBS and HF-rTMS for the treatments of patients with TRD were reported by Fitzgerald et al.’s study (41). Taken together, these findings provide initial support for the role of iTBS as a potential treatment with greater capacity in a shorter stimulation duration for patients with TRD.

As a new form of rTMS, the high-frequency stimulation of iTBS uses 50-Hz triplet bursts that mimic endogenous theta rhythms and influence brain synaptic plasticity more quickly and with longer-lasting effects (42). Previous preclinical studies suggested that the antidepressant effects of iTBS may be related to neuroplasticity (20). Lazzaro et al. (32) found that a 3-min iTBS treatment protocol with 600 pulses per session achieves a similar effect on neural plasticity as the 37.5-min HF-rTMS treatment. Although the recommended iTBS parameters for motor cortex experiments were 600 pulses per session (20), whether it is the optimal dosing strategy for the treatment of TRD is currently unclear. A previous study suggested that increasing the total pulses per session or the number of daily sessions of rTMS may achieve larger antidepressant efficacy (43). In contrast to the standard dose of 600 pulses of iTBS, Li et al. (36) found that a 2-week prolonged iTBS (piTBS) monotherapy with 1800 pulses per session showed the same antidepressant efficacy within a shorter treatment time when compared to the conventional 4–6 week rTMS strategy. However, an exploratory study discovered that doubling the number of iTBS pulses did not enhance the excitatory effect and may have an inhibitory effect (44). Blumberger et al. (45) compared once-daily iTBS and twice-daily iTBS for patients with TRD, finding that using more than 600 iTBS pulses or administering over multiple sessions per day did not produce additional benefits. Interestingly, a recent meta-analysis (5 RCTs, 239 participants) found that active accelerated iTBS (applied 2–10 sessions of iTBS daily treatment with 24,000–90,000 total pulses) achieved a larger response rate in treating major depressive episodes when compared to sham stimulation (46). To date, the heterogeneity of iTBS stimulation parameters such as treatment pulses (600–1800 pulses per session) and stimulation sessions (1–10 sessions per day) has caused some confusion in the clinical practice. Additionally, it is worth noting that prolonging the duration of iTBS stimulation or increasing the number of treatment sessions per day in a patient will be somewhat challenging for the clinical agency. Nevertheless, there is a lack of head-to-head studies comparing the safety and antidepressant effects of iTBS (daily treatment of 600 pulses) with either piTBS or accelerated iTBS for patients with TRD. Thus, further RCTs with high quality are warranted to explore the optimum protocol of iTBS in treating MDD.

Apart from the antidepressant effects, adjunctive TBS may improve the neurocognitive function of psychiatric disorders (47, 48), which has important clinical therapeutic significance. A recent meta-analysis found that iTBS shown a positive effect in enhancing neurocognitive function in healthy adults (49). The findings were consistent with a recent systematic review investigating adjunctive iTBS for neurocognitive dysfunction in elderly patients with schizophrenia (50). However, data on the neurocognitive effects of iTBS versus HF-rTMS were not reported in the two included RCTs (26, 27).

In this systematic review, similar rates of discontinuation and adverse events were observed in the two groups, indicating high clinical acceptability and feasibility of iTBS in the treatment of patients suffering from TRD. This result was consistent with a previous review that reported that iTBS as an add-on therapy was relatively safe for psychiatric disorders and found no serious adverse events except for mild side effects (e.g., headache, dizziness, nausea, and discomfort) (48). Oberman et al. (51) conducted a study focusing on the safety of TBS for the general population and found that only a few subjects suffered from mild adverse events. Similarly, studies focused on other modalities of iTBS, such as piTBS or accelerated iTBS, which were also confirmed to be safe and well tolerated in treating patients with MDD (22, 35, 36).

Overall, the primary strength of this systematic review is that two included RCTs (Jadad score = 5) were classified as high quality. However, there were several limitations in this systematic review that should be noted. First, although a comprehensive systematic search was conducted, only a relatively small number of studies (2 RCTs) met the inclusion criteria for qualitative synthesis. Second, a meta-analysis could not be conducted due to the significant heterogeneity between each included RCT. Third, a medication effect cannot be ruled out because patients remain on their ongoing pharmacological treatment. Fourth, this systematic review only included studies that used the standard dosage of 600 pulses of iTBS for daily treatment, excluding other patterns of iTBS, such as prolonged iTBS and accelerated iTBS. Fifth, all patients in the two included RCTs suffered from treatment-resistant unipolar depression, suggesting that our findings may not be generalizable to treatment-resistant bipolar depression. Finally, this systematic review has not been registered.

## Conclusion

The antidepressant efficacy and safety of iTBS and HF-rTMS appeared to be similar for patients with TRD, although further RCTs with rigorous methodology are needed.

## Data availability statement

The original contributions presented in the study are included in the article/Supplementary material, further inquiries can be directed to the corresponding author.

## Author contributions

X-JL, Q-ML, and Z-JQ selected studies. X-JL and Z-JQ extracted the data. D-BC reviewed all the data and helped mediate disagreements. X-JL and X-HY wrote the first draft. WZ edited the manuscript. All authors contributed to the interpretation of data and approved the last manuscript.

## Funding

This study was funded by the National Natural Science Foundation of China (82101609), Scientific Research Project of Guangzhou Bureau of Education (202032762), Guangzhou Health Science and Technology Project (20211A011045), Guangzhou Science and Technology Project of traditional Chinese Medicine and integrated traditional Chinese and Western medicine (20212A011018), China International Medical Exchange Foundation (Z-2018-35-2002), Science and Technology Program Project of Guangzhou (202102020658), the Science and Technology Program of Guangzhou (2023A03J0839 and 2023A03J0436), Science and Technology Planning Project of Liwan District of Guangzhou (202201012), The Natural Science Foundation Program of Guangdong (2023A1515011383), Guangzhou Municipal Key Discipline in Medicine (2021-2023), Guangzhou High-level Clinical Key Specialty, and Guangzhou Research-oriented Hospital. The funders had no role in study design, data collection and analysis, decision to publish, or preparation of the manuscript.

## Conflict of interest

The authors declare that the research was conducted in the absence of any commercial or financial relationships that could be construed as a potential conflict of interest.

## Publisher’s note

All claims expressed in this article are solely those of the authors and do not necessarily represent those of their affiliated organizations, or those of the publisher, the editors and the reviewers. Any product that may be evaluated in this article, or claim that may be made by its manufacturer, is not guaranteed or endorsed by the publisher.
