# Supplementary material for: Efficacy and safety of intermittent theta burst stimulation versus high-frequency repetitive transcranial magnetic stimulation for patients with treatment-resistant depression: a systematic review
Source: Front Psychiatry. 2023 Jul 31;14:1244289. doi: 10.3389/fpsyt.2023.1244289 (PMC10423820; doi:10.3389/fpsyt.2023.1244289)
Supplement: Supplementary file 1 [file Data_Sheet_1.pdf]

Supplemental Table 1. iTBS versus HF-rTMS for patients with TRD: discontinuation rate and adverse effects

Study	Discontinuation rate (n, %)	iTBS group (n, %)	HF-rTMS group (n, %)	Findings[a]
Blumberger et al., 2018 (Canada)	29 (7)	16 (8)	13 (6)	*P>0.05*
Bulteau et al., 2022 (France)	6 (10)	3 (10)	3 (10)	*P>0.05*
Total	35 (7.4)	19 (7.9)	16 (6.8)	*P>0.05*
**Study**	**Adverse effects**	**iTBS group (n, %)**	**HF-rTMS group (n, %)**	**Findings[a]**
Blumberger et al., 2018 (Canada)	Abnormal sensations	4 (2)	2 (1)	*P>0.05*
	Anxiety or agitation	9 (4)	8 (4)	*P>0.05*
	Back or neck pain	6 (3)	7 (3)	*P>0.05*
	Dizziness	18 (9)	8 (4)	*P>0.05*
	Fatigue	16 (8)	14 (7)	*P>0.05*
	Headache	136 (65)	131 (64)	*P>0.05*
	Insomnia	10 (5)	14 (7)	*P>0.05*
	Migraine aura	4 (2)	3 (1)	*P>0.05*
	Nausea	14 (7)	22 (11)	*P>0.05*

	Tinnitus	3 (1)	1 (<1)	*P>0.05*
	Unrelated accidents	3 (1)	2 (1)	*P>0.05*
	Unrelated medical problem	46 (22)	47 (23)	*P>0.05*
	Vomiting	1 (<1)	1 (<1)	*P>0.05*
Bulteau et al., 2022 (France)	Asthenia	4 (13)	2 (6)	*P>0.05*
	Headache	5 (17)	1 (3)	*P>0.05*

[a] Reflect the differences between iTBS groups and HF-rTMS groups at the treatment endpoints.

Abbreviations: HF-rTMS=high-frequency repetitive transcranial magnetic stimulation; iTBS=intermittent theta burst stimulation; TRD=treatment-resistant depression.

Supplemental Table 2. GRADE Analyses: iTBS versus HF-rTMS for patients with TRD

**Primary/**secondary outcomes	**Study (subjects)**	**Risk of bias**	**Inconsistency**	**Indirectness**	**Imprecision**	**Publication bias**	**Large effect**	**Overall quality of evidence[a]**
**Study defined response**	2 (445)	No[b]	NA	No[c]	No[d]	Undetected	NA	+/+/+/+/; high
**Study defined remission**	2 (445)	No[b]	NA	No[c]	No[d]	Undetected	NA	+/+/+/+/; high
Discontinuation due to any reason	2 (474)	No[b]	NA	No[c]	No[d]	Undetected	NA	+/+/+/+/; high
Abnormal sensations	1 (412)	No[b]	NA	No[c]	No[d]	Undetected	NA	+/+/+/+/; high
Anxiety or agitation	1 (412)	No[b]	NA	No[c]	No[d]	Undetected	NA	+/+/+/+/; high
Asthenia	1 (60)	No[b]	NA	No[c]	Serious[d]	Undetected	NA	+/+/+/-/; moderate
Back or neck pain	1 (412)	No[b]	NA	No[c]	No[d]	Undetected	NA	+/+/+/+/; high
Dizziness	1 (412)	No[b]	NA	No[c]	No[d]	Undetected	NA	+/+/+/+/; high
Fatigue	1 (412)	No[b]	NA	No[c]	No[d]	Undetected	NA	+/+/+/+/; high
Headache	2 (472)	No[b]	NA	No[c]	No[d]	Undetected	NA	+/+/+/+/; high
Insomnia	1 (412)	No[b]	NA	No[c]	No[d]	Undetected	NA	+/+/+/+/; high
Migraine aura	1 (412)	No[b]	NA	No[c]	No[d]	Undetected	NA	+/+/+/+/; high
Nausea	1 (412)	No[b]	NA	No[c]	No[d]	Undetected	NA	+/+/+/+/; high
Tinnitus	1 (412)	No[b]	NA	No[c]	No[d]	Undetected	NA	+/+/+/+/; high
Unrelated accidents	1 (412)	No[b]	NA	No[c]	No[d]	Undetected	NA	+/+/+/+/; high
Unrelated medical problem	1 (412)	No[b]	NA	No[c]	No[d]	Undetected	NA	+/+/+/+/; high
Vomiting	1 (412)	No[b]	NA	No[c]	No[d]	Undetected	NA	+/+/+/+/; high

Abbreviations: HF-rTMS=high-frequency repetitive transcranial magnetic stimulation; NA=not applicable; iTBS=intermittent theta burst stimulation; TRD=treatment-resistant depression; RCTs=randomized controlled trials.

[a] GRADE Working Group grades of evidence: High quality=further research is very unlikely to change our confidence in the estimate of effect. Moderate quality=further research is likely to have an important impact on our confidence in the estimate of effect and could change the estimate. Low quality=further research is very likely to have an important impact on our confidence in the estimate of effect and is likely to change the estimate. Very low quality=we are very uncertain about the estimate.

[b] Two included RCTs were both high-quality randomized double-blind controlled studies. Regarding the random sequence generation, blinding of participants and personnel, incomplete outcome data, and selective reporting were rated as low risk in all RCTs.

[c] Two included RCTs directly compared the efficacy and safety of iTBS with HF-rTMS in patients with TRD.

[d] For dichotomous outcomes, N<300.
